# Design and Tuning of Photoswitches for Solar Energy Storage

**DOI:** 10.3390/molecules26133796

**Published:** 2021-06-22

**Authors:** Raul Losantos, Diego Sampedro

**Affiliations:** Departamento de Química, Centro de Investigación en Síntesis Química (CISQ), Universidad de La Rioja, Madre de Dios, 53, 26006 Logroño, La Rioja, Spain; raul.losantosc@unirioja.es

**Keywords:** energy storage, MOST, computational design, photoswitches

## Abstract

Current energy demand makes it compulsory to explore alternative energy sources beyond fossil fuels. Molecular solar thermal (MOST) systems have been proposed as a suitable technology for the use and storage of solar energy. Compounds used for this application need to fulfil a long series of requirements, being the absorption of sunlight and the energy stored some of the most critical. In this paper, we study different families of well-known molecular photoswitches from the point of view of their potential use as MOST. Starting from basic structures, we use density functional theory (DFT) computational modelling to propose two different strategies to increase the energy difference between isomers and to tune the absorption spectrum. The inclusion of a mechanical lock in the structure, via an alkyl chain and the presence of a hydrogen bonding are shown to directly influence the energy difference and the absorption spectra. Results shown here prove that these two approaches could be relevant for the design of new compounds with improved performance for MOST applications.

## 1. Introduction

The need for alternative energy sources is becoming a main priority due to the high energy demand in industries and transports and the limited availability of fossil fuels [1]. As a society, we have the challenge to find new energy sources environmentally friendly, durable and economic. Solar energy is a potentially unlimited energy source and as such, it has been subject of intense research in the last decades. However, this technology has to face also some drawbacks. Photovoltaic systems have problems to get a continuous energy flow [2]. In addition, solar energy suffers from temporal and seasonal variations. Direct use of solar energy is feasible today, but the energy supply under conditions of low sunlight would be interrupted. This should include night, cloudy days and seasons with decreased sun exposition. In order to solve or minimize this issue, some efforts to store solar energy in different forms have been addressed. A promising alternative is the storage of solar energy as chemical energy, accumulating the energy provided by the sun in form of chemical bonds. Several approaches have been considered, such as photoinduced water splitting [1] or CO and CO_2_ reduction [1]. A different and complementary approach is the use of strongly absorbing compounds capable of using the incident sunlight to perform a photochemical reaction. Among the different available possibilities, molecular photoswitches [3] have been subject to intense research. These systems have been used to control and modify different properties from peptide conformation [4] to liquid crystals structure [5]. These applications rely on light absorption to interconvert the system between two different states and typically, a thermal back reversion occurs at different temperatures. In some cases, when the photoisomer has a high relative energy and it is stable under certain circumstances, the light energy could be partially stored as chemical energy. In turn, this energy could be released with the recovery of the initial, thermodynamically stable isomer of the photoswitch. The overall outcome for this two-step process is the conversion of light energy into heat, which could be released on demand. For this novel application, the energy difference between both isomers is crucial to get a significant energy storage capacity [6]. Also relevant is the ability to release this energy with a controlled trigger. This concept is known as MOST [2], molecular organic solar thermal, and consists of the storage of sun energy in a high-energy isomer for a long time and, eventually, its controlled release to recover the stored energy when required. 

There is a long list of requirements that a molecule should fulfill to be adequate for this technology. Even more, the complex interplay between all the required features for the practical use have prevented the preparation of an ideal candidate within any of the studied types of compounds. Specifically, several factors need to be optimized for a successful implementation of the MOST technology [2]. Among other properties, a high isomerization quantum yield, high-energy storage density, efficient solar spectrum overlap of the parent molecule, long half-life of the high-energy isomer and an efficiently triggered back conversion are key features that should be achieved. Unfortunately, some of these properties are strongly correlated which makes it difficult to optimize the performance in one of these aspects without negatively affecting others. Thus, a good compromise between all these properties should be considered when designing adequate candidates for MOST applications [6,7]. Light absorption and stored energy are two of the most critical properties that need to be fulfilled in this context. In order to be energetically efficient, the molecules designed for this application need to capture as much sunlight as possible. For this, strong absorption in the UV and visible regions is required. In addition, once the photochemical reaction has taken place, the stored energy should be as high as possible. This depends on the molecular weight of the molecule but also on the energy difference between the initial isomer and its photoisomer. In this paper, we will focus on these two critical properties for a series of different photoswitches.

Many different compounds have been proposed for their use as MOST. Azobenzenes [8,9], norbornadienes [10,11], stilbenes [12,13], diruthenium fulvalenes [14,15] and dihydroazulenes [16,17] have been suggested as photoactive systems for this application. Excellent results have been obtained for some of these systems, being norbornadiene and azobenzene derivatives leading examples. While these types of photoswitches have been intensively explored in terms of MOST, related families of compounds have been scarcely assessed as potential candidates for solar energy storage. In this sense, different photoswitches have greatly expanded the design possibilities for different applications, allowing the preparation of an enormous roster of new compounds with promising capabilities to be considered in the development of MOST candidates. 

In the last years, we have been involved in a continuous effort to design [18,19], synthesize [20] and characterize [21] diverse families of photoswitches. The rhodopsin chromophore was used as inspiration in the preparation of a set of switches with very efficient photochemical properties, which could be modulated by quaternization of the nitrogen atom [4]. Oxazolone derivatives were also prepared and their behavior as photoswitches studied [22]. These compounds are very robust and can be easily prepared and functionalized. Bilin, another natural chromophore of phytochromes, was also used as model for the preparation of arylidene-pyrrolinones switches in which we were able to control the most stable isomer through carefully selected substitution [23]. Finally, hydantoin-based switches feature an excellent stability combined with an easy and versatile synthetic route [20]. In addition, azoheteroarenes is a rapidly growing family of switches, which expand the diversity of the azobenzene moiety by modification of the aromatic rings [24].

**Figure 1:**
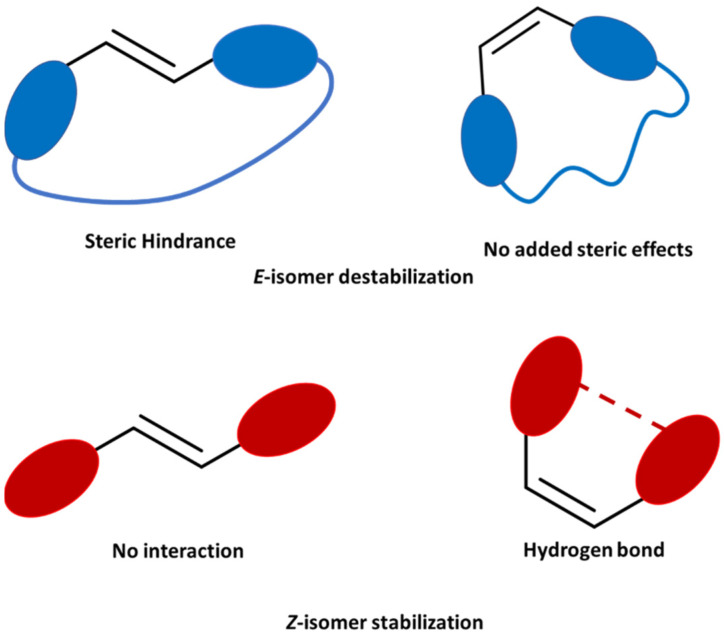
Schematic representation of the proposed strategies to increase the energy storage. (**Top**) In blue the use of a mechanical lock for the increase of the steric hindrance. (**Bottom**) In red, the use of a hydrogen bond to stabilize the *Z*-isomer.

In this work, we evaluate several sets of molecular photoswitches as potential MOST systems. Specifically, we explore the performance of rhodopsin-like, oxazolones, hydantoins, pyrrolinones and azoheteroarenes photoswitches in terms of light absorption and energy storage, two key features for MOST systems. Thus, we explore the potential use of these families of compounds, with a focus on the energy difference between isomers and absorption spectra. We used two different strategies to tune the energy storage (Figure 1). On one hand, we checked the effect of including a mechanical lock, which could alter the relative energy of the two isomers. An alkyl chain connecting the two ends of the structure could greatly modify the stored energy upon photoisomerization by destabilizing the *E* isomer. On the other hand, the inclusion of a hydrogen bond in the structure could also alter the relative energy, in this case by stabilizing the *Z*-isomer. With both strategies, we aim for an increased energy storage, but specifically acting on one isomer or the other. 

We also performed an in-silico screening of a series of azoheretoarenes to identify structural motifs that could lead to an increased energy storage. This family of molecular photoswitches have found extensive use in the last years but their potential as MOST systems has still to be fully evaluated. Finally, some key photophysical properties were also evaluated for selected compounds. To have a practical application as MOST, these compounds should present a strong light absorption in both the UV and visible regions of the spectrum, and a lack of overlap in the absorption of both isomers. In the first case, visible absorption is preferred as it is the main part of the solar spectrum. Thus, to provide an efficient solar energy capture the absorption of the compound should span from the UV well into the visible region. For the second one, the overlap in the absorption of the two isomers should be minimal to avoid side photochemical reactions and competition for the incoming light, which could diminish the efficiency of the process.

## 2. Results and Discussion

A series of compounds for each family was selected based on the synthetic viability and their potential to be used as MOST systems. An initial in-silico evaluation under the framework of the density functional theory (DFT) was performed on different sets of photoswitches (see Computational Section for details). For these switches, different strategies were used to modify the relative energy between isomers (Figure 1). As a first alternative, an alkyl chain connecting both sides of the molecule was included to check the effect of a mechanical lock in the energy difference. For carefully designed structures, this alkyl chain would cause no significant increase in energy in the initial, thermodynamically stable isomer. However, upon irradiation and central double bond isomerization, the photoisomer should suffer from a considerably strain, which will greatly increase its energy and, in turn, the difference in energy between the isomers and the stored energy. The second strategy relies on the stabilization of the initial isomer through hydrogen bonding. The synthetic versatility of the families of switches studied allows for the modification of several points in the structure. Specifically, changing a phenyl ring by a heterocycle capable of H-bonding (pyridine, furan, pyrrole, thiophene) could allow for this interaction in selected compounds (hydantoins, pyrrolinones and oxazolones). The effect of this stabilization in the parent molecule would also increase the energy difference between isomers.

**Figure 2:**
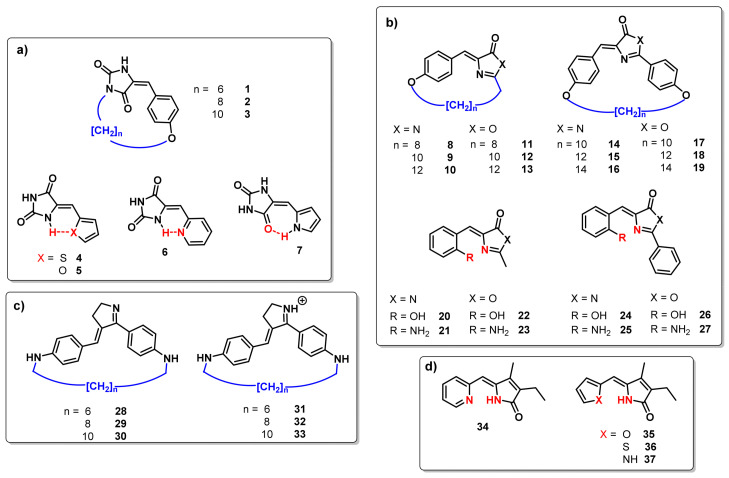
Computational models based on (**a**) hydantoin, (**b**) oxazolone, (**c**) rhodopsin and (**d**) arylidene-pyrrolinone.

Consequently, with both strategies, a series of derivatives were designed to be evaluated through this work. The studied compounds are reported in Figure 2 and can be distributed in different families of photoswitches. Specifically, (Figure 2a) hydantoin, (Figure 2b) oxazolone, (Figure 2c) rhodopsin-based and (Figure 2d) arylidene-pyrrolinone derivatives were studied in terms of energy difference and UV-Vis absorption. 

**Figure 3:**
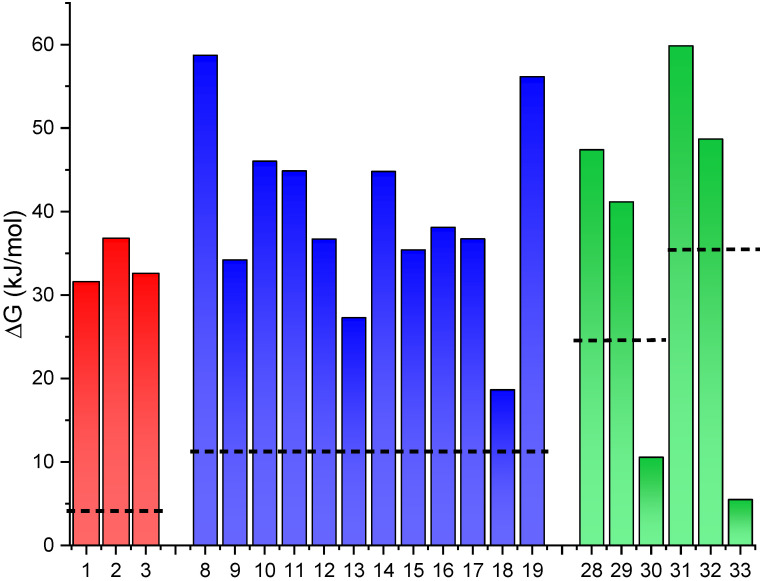
Energy difference between isomers in locked photoswitches (**1***–***3** hydantoins, **8***–***19** oxazolones, **28***–***33** rhodopsin-based neutral and protonated). The dashed line represents the non-restrained system in each case. In **8**–**19** and **28**–**33** the most stable is the *Z*-isomer contrary to the parent molecule, and only in **1**–**3** is the *E*-isomer more stable.

**Figure 4:**
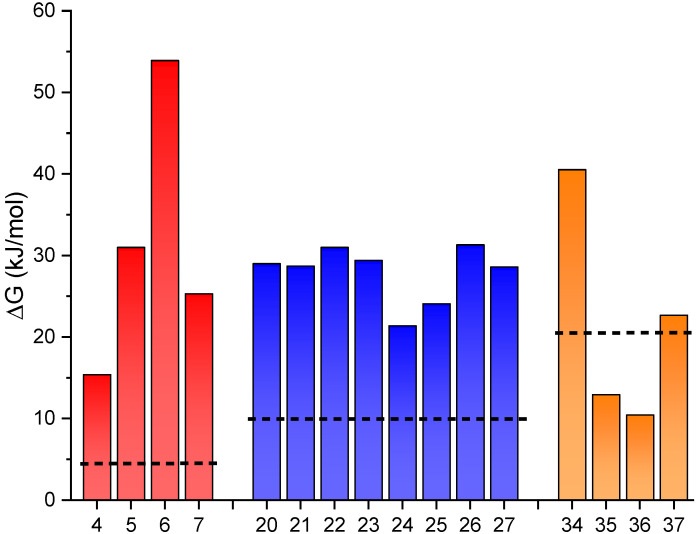
Energy difference between isomers for photoswitches with hydrogen bonds (**4**–**7** hydantoins, **20**–**27** oxazolones, **34***–***37** arylidene-pyrrolinone). The dashed line represents the system without hydrogen bonds (phenyl group) in each case. In all cases the most stable is the *Z*-isomer, and only in compound **7** is the *E*-isomer more stable.

In a first step, all compounds were optimized looking for the most stable conformation of each isomer to obtain the energy difference between *E* and *Z* forms. The computed energy difference can be seen in the following Figure 3 and Figure 4. In these figures, the computed free energy difference between isomers is shown and compared to the non-modified photoswitch (dotted lines for each family of switches). The inclusion of an alkyl chain of different size (Figure 1, top), causes a significant constrain in most of the cases. Only in a few exceptions in which the carbon chain is too long, the stored energy decreases compared to the unmodified switch. It should be noted that for each family of photoswitches, the number of carbon atoms in the chain was adapted according to the end-to-end distance that present the switch without the constraint. Thus, the carbon chain may vary from 6 to 14 to better fit this distance in each case. As a general remark, it is relevant to note the most stable isomer for each family. In the case of hydantoins (**1**–**3**) the non-locked parent molecule presents E as the most stable isomer and this behavior is maintained in the locked compounds, in which the most stable isomer retains the E configuration. Here the energy difference leads to a ten-fold increase applying the mechanical lock strategy, reaching around 35 kJ/mol (Figure 3). However, no significant differences were found between the distinct lengths of the carbon chain used, ranging from 6 to 10 CH_2_ moieties as spacer (compounds **1**–**3**). 

In the case of oxazolidinone and rhodopsin-based photoswitches, the end-to-end distance between the two isomers is different by ca. 2–4 Å. In these cases, the mechanical lock may have a larger effect on the relative energy. Consequently, this modification allows for an increase in the energy difference between isomers close to 60 kJ/mol in some cases (**8** and **19** in oxazolones and **31** in rhodopsin-based). In these families, a different behavior is observed according to the isomer’s stability. In the oxazolone switches, the Z-isomer remains the most stable compound in all cases (locked and non-locked). In contrast, the inclusion of the mechanical lock in case of rhodopsin-based derivatives produces a change in the relative stability becoming the *Z*-isomer the most stable instead of the *E*-isomer, which is the most stable form in non-restricted compounds. Looking in more detail into the differences observed in **8** and **19** with respect to other compounds, a clear effect is observed here. In **8**, the small carbon chain strongly destabilizes the *E*-isomer while it does significantly affect the *Z*-isomer due to its smaller end-to-end distance. On the other hand, the longer carbon chain of **19** destabilizes both isomers, with a greater effect over the *E*-isomer as this alkyl chain fits better the distance in the *Z*-isomer. The same effect is observed for **31**, producing an increase value for the stored energy with respect to the neutral form of the switch. In case of the longest carbon chain for retinal, **30** and **33**, the presence of 10 CH*_2_* moieties yields in an excessive space which modifies both isomers decreasing the energy gap. 

As the main conclusion of this section, the addition of a conformational restrain to the switches can induce a relative destabilization on one of the isomers, thus altering the energy difference and the stored energy. 

The second strategy consisting of the effect of hydrogen bonds was evaluated in different switches by inclusion of common heterocycles like thiophene, furan, pyridine and pyrrole. For that, several derivatives (**4**–**7**, **20**–**27** and **34**–**37**) were considered. The effect of this hydrogen bond in the molecular geometry and, in turn, in the energy difference was appreciated in most of the cases. According to the bond strength, the reduction in the distance between X-H implies the stabilization of the isomer. Best results were found for **6**, in which the energy difference between isomers reached 54 kJ/mol, as can be seen in Figure 4. The same strategy was used in the case of phytochrome derivatives (**34**–**37**). Here again, a correlation between hydrogen bond strength and stabilization of the initial isomer was found, increasing the relative energy difference to 44 kJ/mol for **34**. In compounds **35** and **36** a more planar geometry respect to the parent phenyl-substituted molecule yields in a moderate stabilization of *E*-isomer. This effect is almost negligible in terms of enthalpy difference as can be seen in the ESI (Supplementary Materials). A similar strategy was used in case of oxazolone derivatives to facilitate the formation of hydrogen bond. In this case, the modification consists of the addition of a hydroxyl or amino group in the ortho position of the phenyl ring which can interact with the nitrogen of the oxazolidinone. For that, switches based on oxazolidinone (**20**–**27**) displays a moderate increase in the stored energy from 10 to ca. 30 kJ/mol. 

Interestingly, as shown in Figure 3 and Figure 4, both to affect the stored energy of these switches for a potential use as MOST strategies are promising. This could allow for the rational design and the future preparation of selected compounds within any of these families as candidates for energy storage. The synthesis, photoisomerization mechanism, stability, photophysical properties, fatigue resistance and many other features have been already assessed for many different compounds of these types [4,20,22,23]. Thus, these strategies could be useful to try to specifically optimize the stored energy in selected compounds with already a good list of requirements fulfilled.

**Figure 5:**
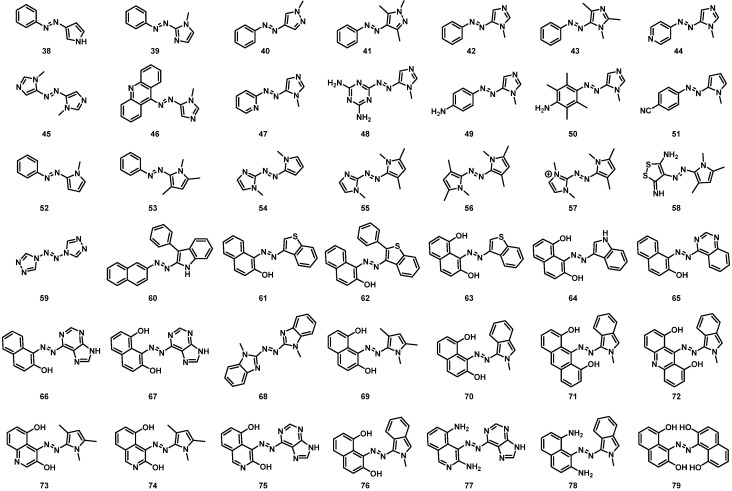
Azoheteroarene derivatives studied.

**Figure 6:**
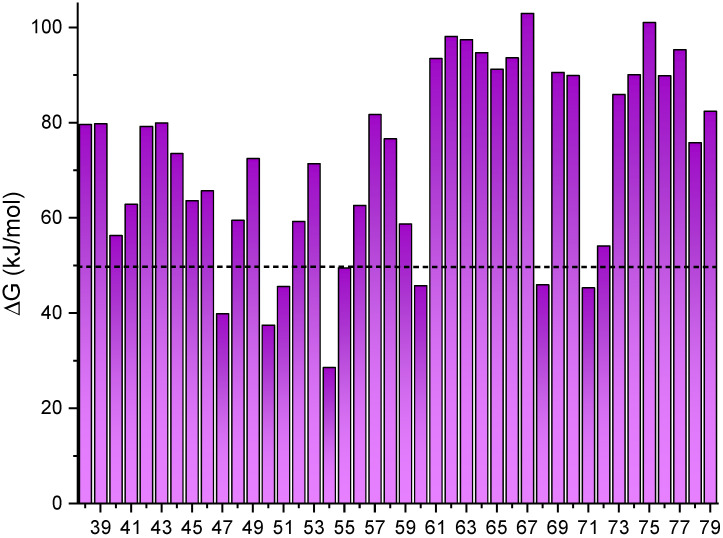
Energy difference achieved with different azoheteroarenes. The dashed line represents azobenzene. In all cases the most stable one is the *E*-isomer.

As previously mentioned, azoheteroarene derivatives are attracting increasing interest in the last years due to the wide structural available landscape that could be provided by changing the phenyl rings in azobenzene by different heterocycles. However, these compounds have been scarcely considered as MOST candidates. In the following, we will focus on the influence of different heteroarene moieties in the energy storage. For this, a series of 42 azoheteroarenes (**38**–**79**, Figure 5) with a comprehensive range of aromatic rings was computed to evaluate the energy difference between isomers and the UV-Vis spectrum. This would allow to estimate the ability of this family of compounds in two critical features for MOST such as energy storage and light absorption. The computed compounds were selected to provide a general overview of the behavior of this family of switches while testing the hydrogen bond strategy that has been shown above to efficiently affect the energy storage. Results for the energy difference are shown in Figure 6.

Detailed analysis of the computational results allowed the extraction of some interesting observations. Compounds including the 2-hydroxynaphtalene or 2,8-dihydroxynaphtalene (**63**–**67**) and 2-N-methylpyrrole-based (**52**–**53**) moieties increases the energy difference close to 90 and 70 kJ/mol in average, respectively, for these substituents. In contrast, smaller differences were found for switches with two small cycles, like **47** and **54**. 

**Figure 7:**
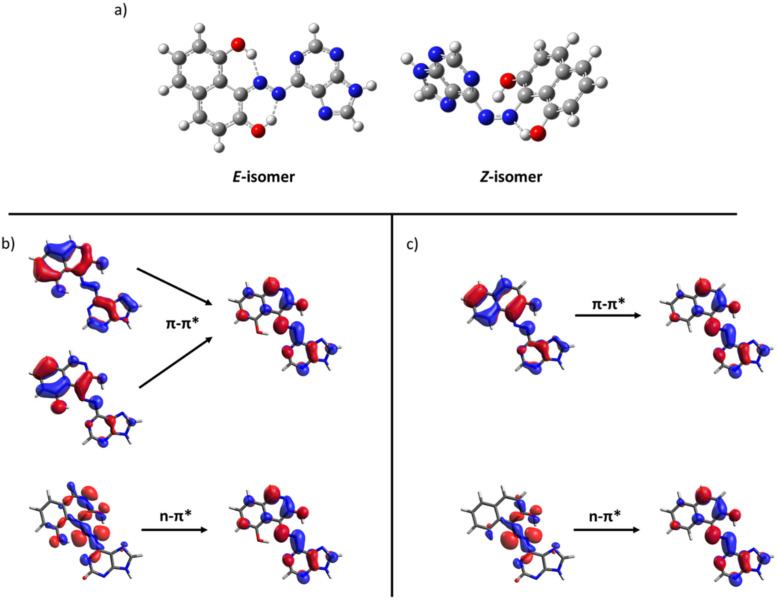
(**a**) Optimized structures of the *E* and *Z*-isomers of **67**. Representative orbitals involved in the excitation of azoheteroarenes **66** (**b**) and **75** (**c**).

Due to the wide structural variability of the compounds studied, it may be misleading to focus on one specific example. Instead, general conclusions could be drawn by comparing groups of compounds sharing the same moiety. First, an increase in the steric hindrance causes variations in the energy difference. For instance, comparing **49** and **50**, the additional methyl groups cause a more rotated *Z*-isomer which reduces the energy difference. Also, the presence of hydroxyl groups in specific positions may allow the formation of a hydrogen bond with the azo group nitrogen atoms. As seen above, this kind of interaction may greatly affect the relative energy. In this case, the E-isomer is stabilized, increasing the energy difference. This behavior was found in several compounds including the 2,9-dihydroxynaphtalene moiety. As an example, Figure 7 shows the geometries for the *E* and *Z* isomers of **67**. The hydrogen bonds in the *E*-isomer (Figure 7, left) contribute to decrease the energy for this isomer and a consequent increase in the energy difference (108 kJ/mol, Figure 6). Finally, the inclusion of nitrogenated aromatic rings (imidazole, such as **39** and **44**) seems to increase the energy difference compared with the standard azobenzene.

**Figure 8:**
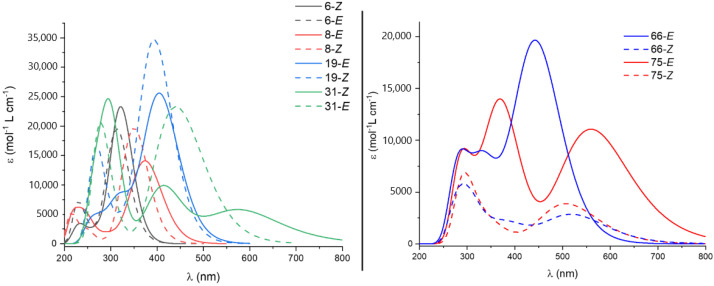
UV-Vis spectra for selected compounds of (**left**) different types of photoswitches (**6**, **8**, **19** and **31**) and (**right**) azoheteroarenes (**66** and **75**).

Once we have shown through computational methods that the stored energy could be increased by careful selection of the structure, we focused on another important feature for MOST, the UV-Vis absorption spectrum. Spectra were computed for all the compounds using time-dependent DFT methods (see Computational Details). For this, the most promising candidates from the point of view of energy difference across the different families already introduced can be seen in Figure 6. There, the absorption spectra of both isomers are plotted for the slected compounds for different families of switches: hydantoins (**6**), imidazolones (**8**), oxazolones (**19**), rhodopsin-based switches (**31**) and azoheteroarenes (**66** and **75**). In all cases except azoheteroarenes, the non-modified compounds feature relevant absorption only in the UV region and a small shift between isomers, ca. 10 nm, in agreement with experimental results [4,20,22,23]. As explained in the Introduction, these two issues clearly reduce the applicability of these compounds as MOST systems. Thus, the inclusion of different carbon chains to affect the relative energy of the isomers slightly increases the gap but has a small impact on the optical properties in these photoswitches. As can be seen in Figure 8, left, only **31** presents a noticeable change in the spectral overlap for both isomers. This fact is due to the high constrain that imposes the presence of a short alkyl chain (six carbon atoms) in the chromophore, which even inverts the stability order of the isomers, as discussed previously. In contrast with the other families, computed azoheteroarenes present strong absorptions in the visible region and, in some cases, an appropriate shift between isomers is found minimizing the spectral overlap. Both **66** and **75** feature absorption spectra with a significant overlap with sunlight, as can be seen in Figure 8, right. Looking further into the nature of the excited states participating in the absorption process of the stable isomer, the involved orbitals can be seen in Figure 7 for these two compounds. As can be seen, the n orbital is clearly stabilized due to the hydrogen bond with the hydroxyl group. Accordingly, the low-lying n-π* transition appears notably blueshifted respect to the brighter π-π* transition of the *E*-isomer. Compounds with the 3,5-dihydroxy-isoquinoline moiety slightly blueshift the n-π* transition of the *Z*-isomer occurring at higher energies than the π-π* transition of the *E*-isomer. 

From this computational screening some design hints could be extracted as general conclusions. The hydrogen bond formation also plays a relevant role in the azoheteroarenes blueshift for the undesired n-π* transition beyond the increase in the energy difference which confirm some of the described azoheteroarenes as potential candidates for MOST systems. 

**Figure 9:**
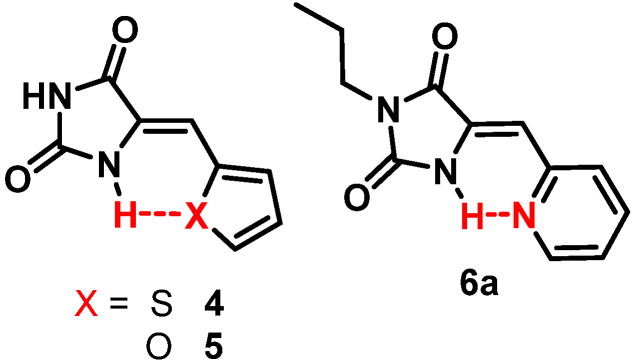
Hydantoin derivatives prepared experimentally.

The design concepts unveiled in the computational study where further tested through the preparation of some selected compounds. For this, we used the hydantoin-based photoswitches as this family combines promising results from the computational study and a versatile synthetic route [20]. Also, due to the increased energy storage values obtained using the hydrogen bond strategy (up to 54 kJ/mol for **6**, Figure 4) compared with the mechanical lock (up to 36 kJ/mol for **2**, Figure 3), we focused on the preparation of compounds **4–6** (see Figure 9) as proof of concept of this strategy. 

Compounds **4** and **5** were made following the previously reported method [20] using a suspension of hydantoin and the desired aldehyde in acetic acid as solvent and NH_4_AcO as catalyst at 120 °C for 12 h. In case of **6**, this reaction does not produce the desired product. Instead, **6a** could be prepared through a modification including a prior alkylation step and the synthesis assisted by microwave and basic media, as described in literature [25]. This procedure affords the *Z*-isomer in all cases for compounds **4**–**6a**, due to the greater thermodynamic stability of this isomer expected from the presence of hydrogen bonding.

All compounds were photochemically characterized with UV-Vis spectra and irradiated under different light sources to find the photostationary state (PSS) for each derivative. UV-Vis data recorded in Table 1 were collected over 5E-5 M solutions in acetonitrile. PSSs were calculated by NMR integration with the vinylic proton signal for both isomers in d_6_-DMSO. Irradiation were done using a 400 W Pyrex-filtered medium-pressure Hg lamp or a LUZCHEM photoreactor with UVA lamps with an emission wavelength centered in 350 nm (14 lamps *×* 8 W/lamp).

All compounds show a similar spectrum in the UV-Vis region, with a maximum in all cases centered between 330 and 350 nm, which is in good agreement with the computational data also validating the computational method used. Irradiation with 350 nm light improves isomerization efficiency between 20–40%. In the case of **4** and **5** the presence of methanol does not improve the isomerization weakening the hydrogen bond, that effect is partially observed in **6a**, which can switch 5% in methanol and no rotation is observed in DMSO. 

## 3. Materials and Methods

### 3.1. Computational Details

All calculations were performed under the framework of the density functional theory (DFT) as implemented in the Gaussian16 package. For that, the B3LYP functional was used together with the 6–31G* basis set which is an extensively used methodology for organic compounds. All critical points obtained were characterized with frequency calculations to include the ZPE and free energy corrections and verify the stationary points as minima (zero imaginary frequencies) in all cases. Free energy differences were calculated by difference between the obtained free energy corrections of both isomers in each case. The time-dependent density functional theory was used to compute the absorption spectra for each compound. For that, the UV-VIS spectra were computed in vacuum at the B3LYP/6–31G* level considering ten roots and the spectra were plotted as a convolution of Gaussian functions. 

### 3.2. General Information

All starting materials sourced from commercial suppliers were used as received unless otherwise is stated. UV-Vis spectra were recorded using an Ocean optics USB4000 spectrometer and quartz cuvettes.

### 3.3. General Synthesis Procedure 

A solution of imidazolidine-2,4-dione (2.16 g, 20 mmol) and ammonium acetate (276 mg, 3.6 mmol) in acetic acid (5 mL) was added to aldehyde (20 mmol), and the resulting mixture was stirred at 120 °C for 16 h. After cooling, the reaction mixture was poured in water. The resulting precipitate was filtered and dried affording the 5-arylideneimidazolidine-2,4-diones as pale color solids (with yields ranging from 75 to 90%) [20].

Compound **4** was obtained according to the general procedure as a brownish solid with a yield of 80%. ^1^H-NMR (300 MHz, DMSO-d_6_) δ ppm 11.25 (s, 1H), 10.34 (s, 1H), 7.70 (dd, *J* = 5.0, 1.0 Hz, 1H), 7.60 (dt, *J* = 3.7, 0.9 Hz, 1H), 7.17 (ddd, *J* = 5.1, 3.7, 0.5 Hz, 1H), 6.57 (s, 1H). ^13^C-NMR (75 MHz, DMSO-d_6_) δ ppm 165.2, 155.4, 136.0, 129.0, 128.8, 128.6, 126.2, 101.5. UV-Vis (CH_3_CN): λ (nm) = 333, 348 (ε = 5200 M^−1^cm^−1^). ES-MS (+) (C_8_H_6_N_2_O_2_S + H): calc. 195.0223 found 195.0224.

Compound **5** was obtained as a brownish solid according to the described procedure with 75% yield. ^1^H-NMR (300 MHz, methanol-d_4_) δ ppm 7.69 (d, *J* = 1.4 Hz, 1H), 6.72 (dd, *J* = 3.5, 0.6 Hz, 1H), 6.56 (dd, *J* = 3.5, 1.8 Hz, 1H), 6.43 (s, 1H). ^13^C-NMR (75 MHz, methanol-d_4_) δ ppm 167.0, 156.8, 151.3, 146.0, 127.2, 114.9, 113.4, 99.0. UV-Vis (CH_3_CN): λ (nm) = 335, 350 (ε = 4800 M^−1^cm^−1^). ES-MS (+) (C_8_H_6_N_2_O_3_ + H): calc. 179.0451.0223 found 179.0449.

The preparation of **6a** has been already reported [25].

## 4. Conclusions

In this work, some families of *E/Z*-photoswitches were computationally explored in terms of energy storage and in the most interesting cases, the UV-Vis absorption properties were simulated. With this large screening, several strategies to increase the energy difference between isomers were tested, finding that the formation of hydrogen bonds and the inclusion of a mechanical lock by a carbon chain are relevant strategies. Results showed a very relevant increase in the energy difference from the previously described compounds. For the hydantoin, rhodopsin, oxazolidinone and phytochrome switches the energy difference is increased, but their UV absorption and a small shift in the absorption spectra between isomers could hamper their use as MOST candidates. In contrast, several examples of the proposed azoheteroarenes reach energy differences that make these compounds potentially relevant. This fact, in combination with the large spectral overlap found with sunlight highlight these derivatives as possible candidates for their use in MOST devices. 

## Figures and Tables

**Table 1:** Absorption spectra and photostationary state (PSS) in different irradiation conditions. ^a^ Value in nm for Z-isomer. ^b^ Using a 400 W Pyrex-filtered medium-pressure Hg lamp. ^c^ Using a photoreactor at 350 nm. ^d^ Using photoreactor at 350 nm with d_4_-methanol solutions.

Compound	λ_max_^a^	PSS (*Z/E*) ^b^	PSS (*Z/E*) ^c^	PSS (*Z/E*) ^d^
**4**	333, 348	55/45	27/73	27/73
**5**	335, 350	63/37	43/57	43/57
**6a**	328, 343	100/0	100/0	95/5

## Supplementary Materials

The following are available online. Enthalpy energy differences, Characterization details, NMR spectra. Cartesian coordinates are available on demand.
