# Supplementary material for: Design and Tuning of Photoswitches for Solar Energy Storage
Source: Molecules. 2021 Jun 22;26(13):3796. doi: 10.3390/molecules26133796 (PMC8270257; doi:10.3390/molecules26133796)
Supplement: Supplementary file 1 [file molecules-26-03796-s001.zip › molecules-1257353-supplementary.pdf]

Electronic Supplementary Information

Design and tuning of photoswitches for solar energy storage

Raúl Losantos [1] and Diego Sampedro [1,*]

[1] Departamento de Química, Centro de Investigación en Síntesis Química (CISQ), Universidad de La Rioja, Madre de Dios, 53, 26006 Logroño, La Rioja, Spain. E-mail: diego.sampedro@unirioja.es

Contents

1. Characterization methods
2. NMR spectra

1. Characterization methods

^{1}H and ^{13}C NMR spectra were recorded on a Bruker ARX-300. Dimethylsulfoxyde-d_6 and methanol-d_4 have been used as deuterated solvents, using its signal as standard. Chemical shifts are given in ppm and coupling constants in hertz.

Electrospray mass spectra were recorded on an Ultra Performance Liquid chromatography Acquity Waters with an interface to high resolution mass spectrometry was performed in a Bruker Microtof-Q electrospray source in positive-ion mode.

2. NMR spectra

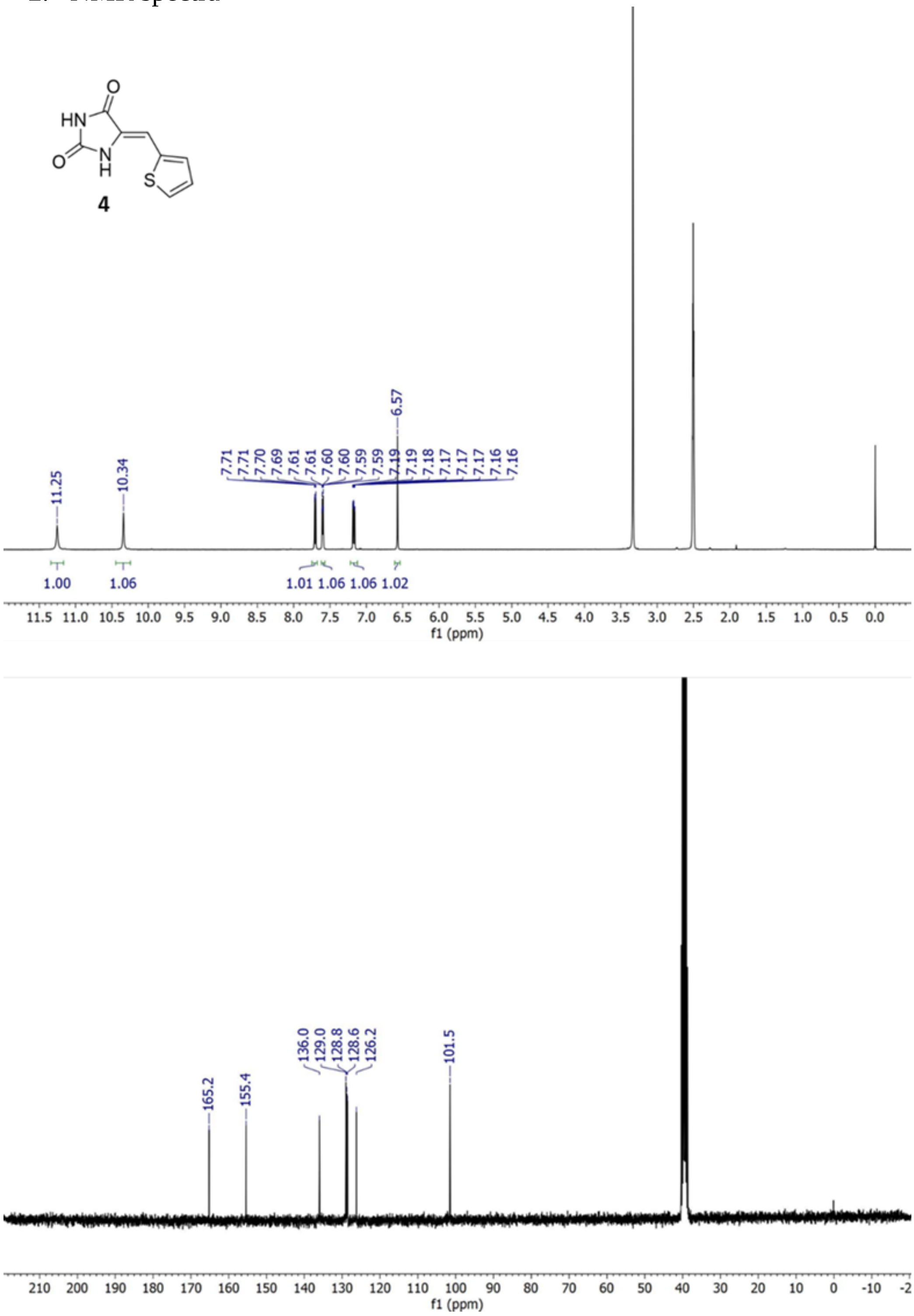

Figure SI1. NMR spectra of **4**.

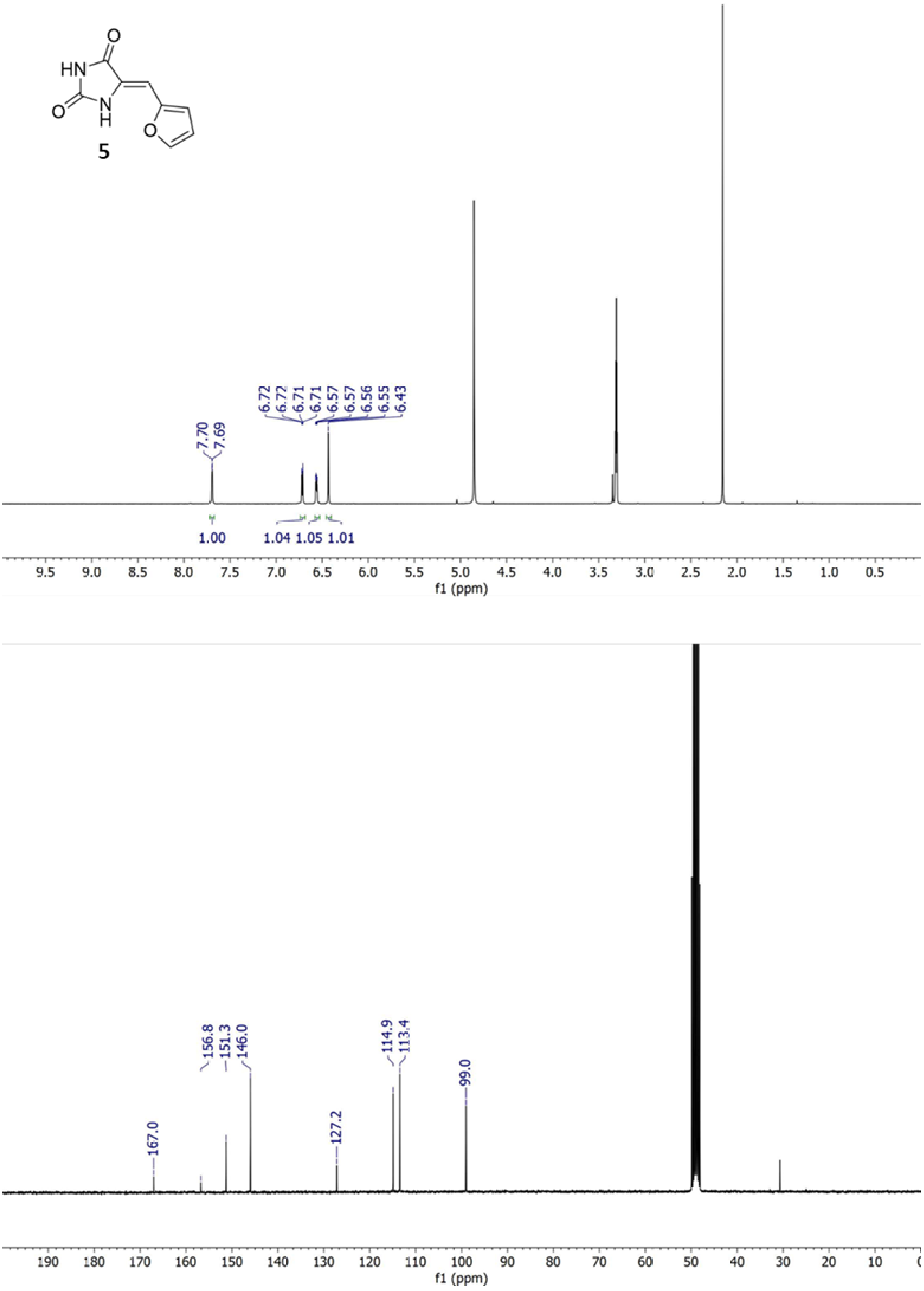

Figure SI2. NMR spectra of **5**.
